# Supplementary material for: Clusterin knockdown sensitizes prostate cancer cells to taxane by modulating mitosis
Source: EMBO Mol Med. 2016 May 19;8(7):761–78. doi: 10.15252/emmm.201506059 (PMC4931290; doi:10.15252/emmm.201506059)
Supplement: Supplementary file 1 — Expanded View Figures PDF [file EMMM-8-761-s001.pdf]

Expanded View Figures

Figure EV1. Clusterin interaction with Cdc25C affects cell function.

A Gene clusters identified by differential expression profiling of RNA microarray analysis comparing PC3 cells transfected with siSCR versus siCLU. Data were analyzed through the use of QIAGEN's Ingenuity Pathway Analysis (IPA; QIAGEN, Redwood City) (www.quiagen.com/ingenuity). ($P < 0.05$; right-tailed Fisher's exact test).

B *In silico* correlation analysis of Cdc25C and CLU mRNA levels in 460 prostate cancer patients using GeneSapiens data set http://ist.medisapiens.com/ (Spearman correlation: $r = -0.23$; $P < 0.001$).

C Co-immunoprecipitation from parental PC3 cells. Proteins were immunoprecipitated with specific anti-Cdc25C and CLU antibodies and analyzed by immunoblotting. Actin and IgG were used as negative controls.

D Duolink proximity ligation assay between Cdc25C and CLU in PC3 cells. Confocal microscopy was used to detect the interaction (red dots). DNA was counterstained with DAPI (blue). Actin and eIF4G were used as a negative control. Scale bar represents 10 μm.

E FACS analysis showing percentage of the PC3 cells positive for phosphohistone H3 after treatment with siCLU compared to siSCR. Error bar represents mean ± SEM, $n = 3$, ***$P < 0.0001$ by the Mann–Whitney test.

F Western blot analysis for CLU, Cdc25C, Cdc25C-T48, and pERK antibodies in PC3 cells after transfection with siSCR or siCLU, followed by treatment with or without MAPK inhibitor UO126, synchronization and nocodazole release at the indicated time. Vinculin was used as loading control.

© 2016 The Authors

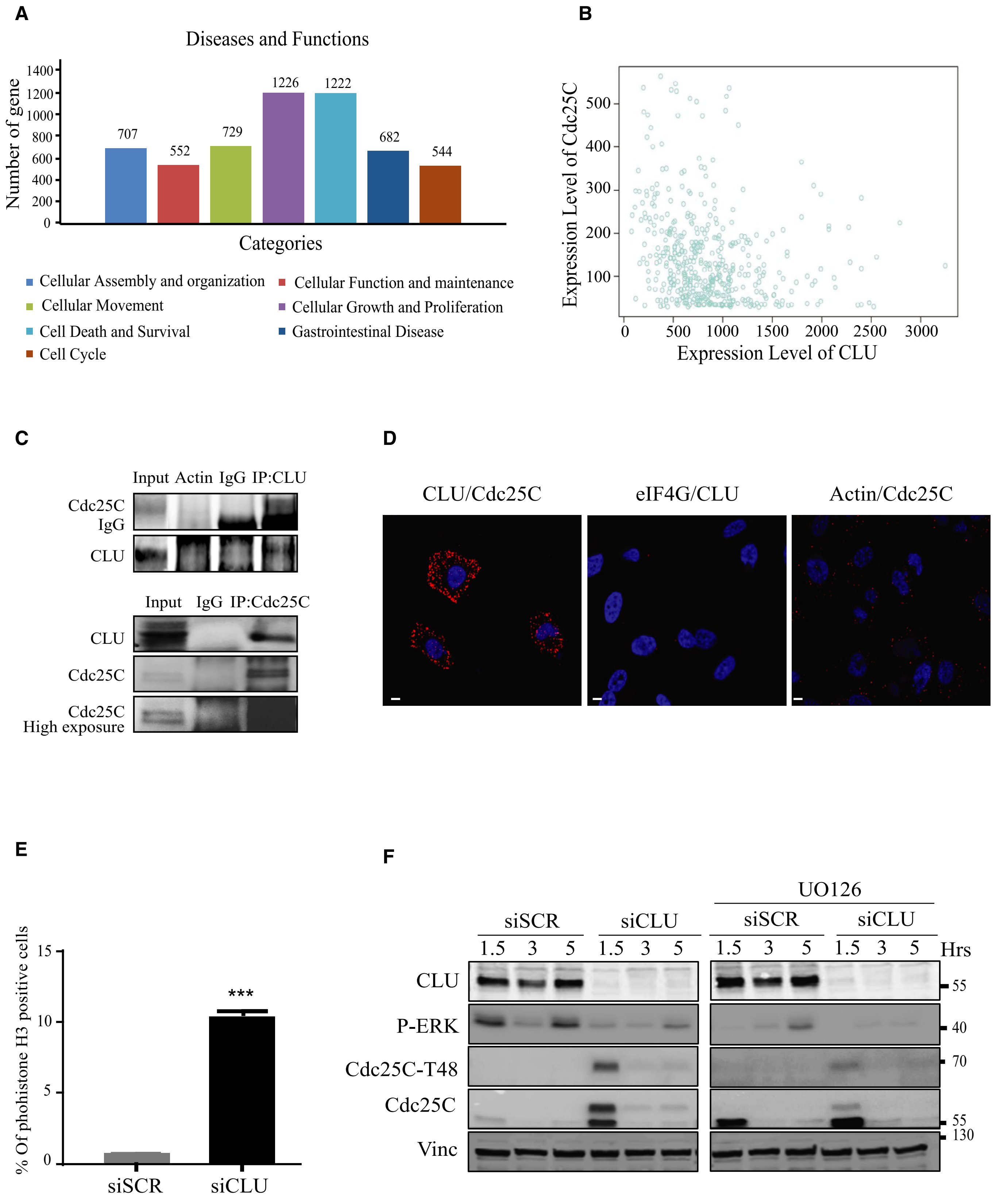

Figure EV1.

 © 2016 The Authors

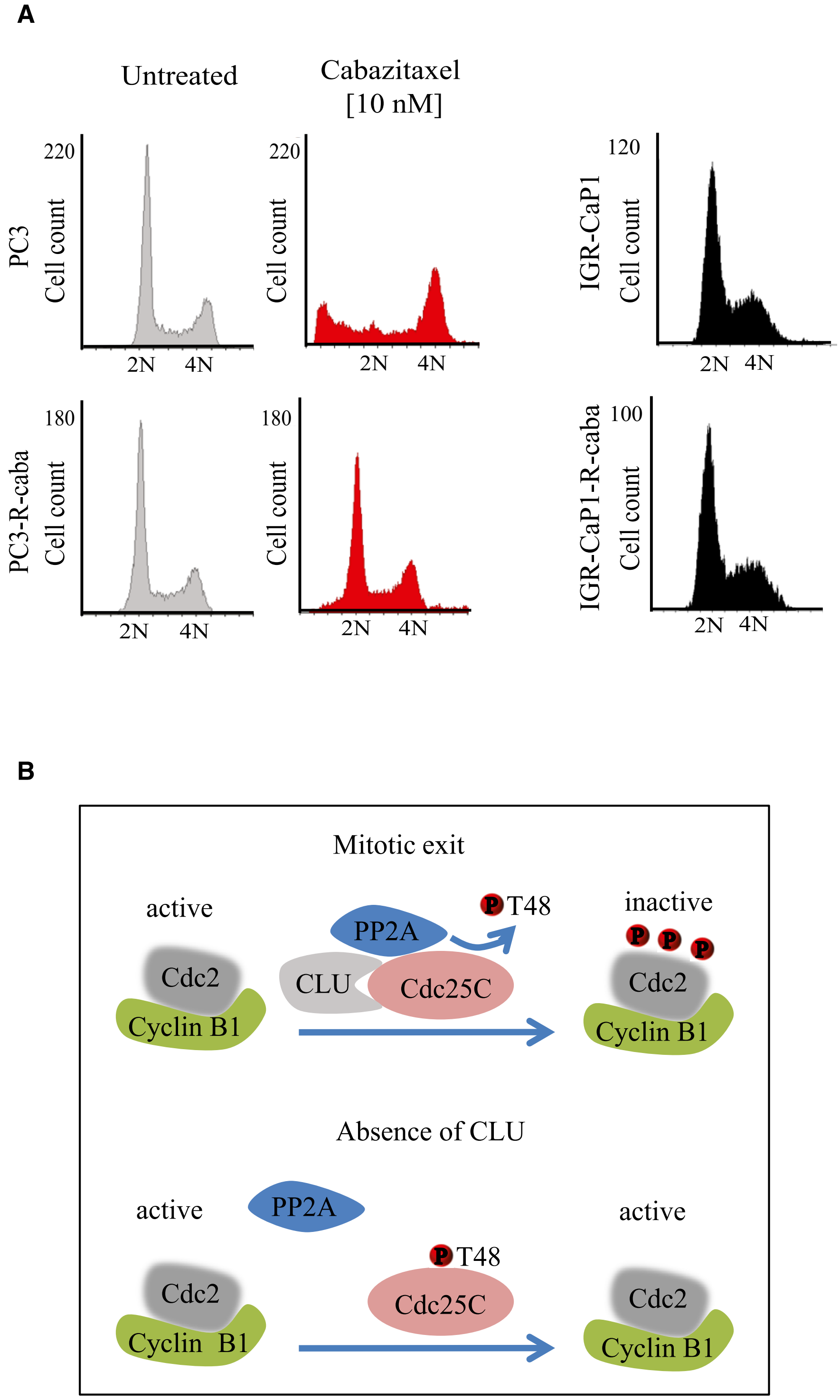

Figure EV2. CLU regulates PP2A dephosphorylation of Cdc25C and exit from mitosis.

A Cell cycle profile of IGR-CaP1, IGR-CaP1-R-caba; and PC3, PC3-R-caba cells in the presence or absence of 10 nM cabazitaxel.

B Schema of molecular interaction between CLU and Cdc25C that regulate Cdk1/cyclin B1 activity.

© 2016 The Authors
