# Supplementary figures and images for: Clusterin knockdown sensitizes prostate cancer cells to taxane by modulating mitosis
Source: EMBO Mol Med. 2016 May 19;8(7):761–78. doi: 10.15252/emmm.201506059 (PMC4931290; doi:10.15252/emmm.201506059)

Right panels

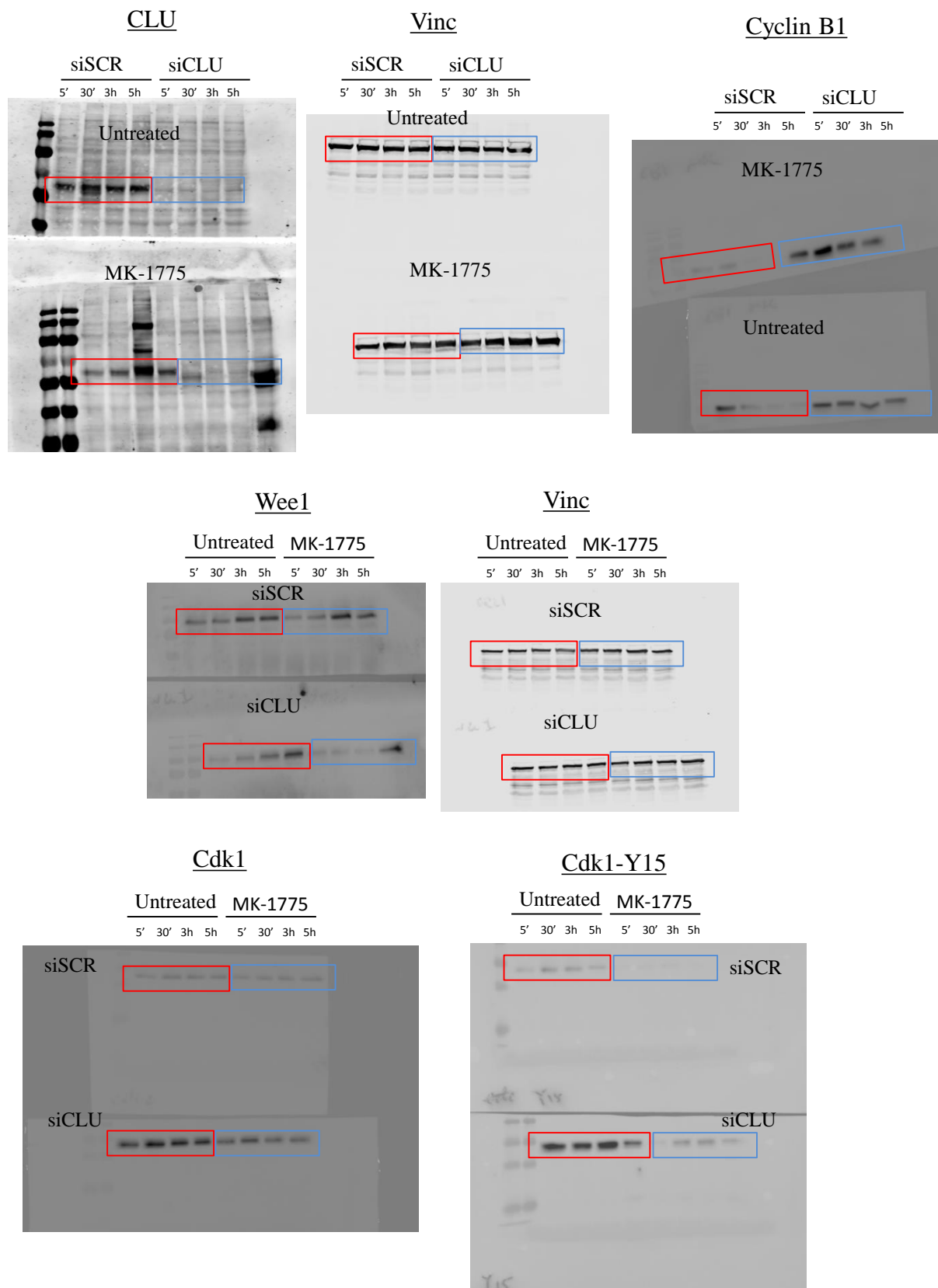

Supplement: Supplementary file 3 — Source Data for Figure 7 [file EMMM-8-761-s002.pdf]
